# Rice Sheath Rot: An Emerging Ubiquitous Destructive Disease Complex

**DOI:** 10.3389/fpls.2015.01066

**Published:** 2015-12-11

**Authors:** Vincent de P. Bigirimana, Gia K. H. Hua, Obedi I. Nyamangyoku, Monica Höfte

**Affiliations:** ^1^Laboratory of Phytopathology, Department of Crop Protection, Faculty of Bioscience Engineering, Ghent UniversityGhent, Belgium; ^2^Department of Crop Science, School of Agriculture, Rural Development and Agricultural Economics, College of Agriculture, Animal Science and Veterinary Medicine, University of RwandaMusanze, Rwanda

**Keywords:** rice, sheath rot, *Sarocladium oryzae*, *Pseudomonas fuscovaginae*, *Fusarium fujikuroi* complex, fumonisins, grain discoloration, phytotoxins

## Abstract

Around one century ago, a rice disease characterized mainly by rotting of sheaths was reported in Taiwan. The causal agent was identified as *Acrocylindrium oryzae*, later known as *Sarocladium oryzae*. Since then it has become clear that various other organisms can cause similar disease symptoms, including *Fusarium* sp. and fluorescent pseudomonads. These organisms have in common that they produce a range of phytotoxins that induce necrosis in plants. The same agents also cause grain discoloration, chaffiness, and sterility and are all seed-transmitted. Rice sheath rot disease symptoms are found in all rice-growing areas of the world. The disease is now getting momentum and is considered as an important emerging rice production threat. The disease can lead to variable yield losses, which can be as high as 85%. This review aims at improving our understanding of the disease etiology of rice sheath rot and mainly deals with the three most reported rice sheath rot pathogens: *S. oryzae*, the *Fusarium fujikuroi* complex, and *Pseudomonas fuscovaginae*. Causal agents, pathogenicity determinants, interactions among the various pathogens, epidemiology, geographical distribution, and control options will be discussed.

## Introduction

Rice is an important crop worldwide, serving as the staple food for half of humanity and additionally being used in industry and for animal feed. Rice is grown in various agro-ecological zones in tropical and subtropical areas, especially in Asia, the continent accounting for 90% of the world production (IRRI, 2015a). It faces many production constraints, including pests and diseases.

The major feature of rice sheath rot disease is rotting and discoloration of the sheath, leading to chaffiness and sterility of resulting grains. For many years, rice sheath rot was considered as a minor and geographically limited disease. It is only recently that it gained momentum and became widespread. Since the green revolution in Asia in the 1960s, there have been substantial changes in rice farming systems: use of high yielding varieties, increased use of fertilizers, efficient systems of water use, seeding methods, etc. Crop intensification practices such as increased plant density, a high rate of nitrogen fertilizers and the use of semi-dwarf and photoperiod-insensitive cultivars, favor the susceptibility of rice to some diseases and the sheath rot complex is one of them. It is hypothesized that the new photoperiod-insensitive cultivars have lost the capacity of avoiding flowering under conditions of high humidity and high temperature, conditions that are conducive to effective disease attacks (Mew et al., 2004b). Additionally, the last decades saw the boosting of international exchange of planting materials which may have contributed to the spread of the disease.

Rice sheath rot is a disease complex that can be caused by various fungal and bacterial pathogens. Major pathogens associated with rice sheath rot are fungi such as *Sarocladium oryzae* and *Fusarium* sp. belonging to the *Fusarium fujikuroi* complex and the bacterial pathogen *Pseudomonas fuscovaginae*. A variety of other pathogens have been associated with rice sheath rot. An overview is given in **Table 1**. The various described sheath rot agents all cause very similar disease symptoms (Cottyn et al., 1996a). This explains why there are practically no comprehensive studies mentioning the link between the presence and quantity of disease inoculum and yield loss (Mew and Gonzales, 2002). The unpredictable nature of the factors acting in the pathosystem explains why losses attributed to *S. oryzae* can be as variable as in the range of 20–85% (Sakthivel, 2001).

**Table 1:** Organisms associated with rice sheath rot.

Causal agent	Taxonomic position	Synonyms or other used names	Occurrence	Geographic distribution	Reference
**Fungi**					
*Sarocladium oryzae*	Ascomycota, Hypocreales	*Acrocylindrium oryzae, Cephalosporium caerulans*, *Sarocladium attenuatum*	Lowland (<1250 m)	32 countries	Purkayastha and Ghosal, 1985; Sakthivel, 2001; Bills et al., 2004; Giraldo et al., 2015
*Gibberella fujikuroi* complex	Ascomycota, Hypocreales	*Fusarium fujikuroi, F. proliferatum, F. verticillioides, F. moniliforme*	Ubiquitous	Everywhere	Desjardins and Plattner, 1997; Abbas et al., 1998; Kushiro et al., 2012; Quazi et al., 2013; Aoki et al., 2014
*Fusarium graminearum*	Ascomycota, Hypocreales	*F. zeae*	5–30°C (optimum around 15°C), high relative humidity	Everywhere where temperatures are low and humidity is high	Singh and Devi, 1990; Naeimi et al., 2003; Goswami and Kistler, 2004; Leplat et al., 2012; Aoki et al., 2014; Backhouse, 2014
*Fusarium incarnatum-equiseti* complex	Ascomycota, Hypocreales	*F. equiseti*	Found in regions with cool through to hot and arid climates	Mainly in wheat-growing areas	Fisher and Petrini, 1992; Wheeler et al., 1999; Marín et al., 2012
*Fusarium oxysporum* complex	Ascomycota, Hypocreales	–	Ubiquitous	Nepal, Italy	Fisher and Petrini, 1992; Abbas et al., 1995; Desjardins et al., 2000; Ruiz-Roldán et al., 2015
*Cochliobolus lunatus*	Ascomycota, Pleosporales	*Curvularia lunata*	Wide host range and common in paddy fields	India, Bangladesh, China	Lakshmanan, 1992, 1993a; Shamsi et al., 2003; Liu et al., 2009; Gao et al., 2015
*Gaeumannomyces graminis*	Ascomycota, Incertae sedis	*Ophiobolus oryzinus*	Wind is an important dissemination factor; found in tropical, subtropical and southern temperate climates	South and North America, Australia	Walker, 1972; Gnanamanickam and Mew, 1991; Frederick et al., 1999; Elliott, 2005; Peixoto et al., 2013
*Sclerotium hydrophilum*	Basidiomycota, Cantharellales	*Ceratorhiza* sp.	Infection on aquatic or semi-aquatic plants of wet meadows and marshes	Australia	Lanoiselet et al., 2002; Yang et al., 2007; Hu et al., 2008; Xu et al., 2010
*Sclerotium oryzae*	Basidiomycota, Agaricales	*Ceratobasidium oryzae-sativae*	Overwintering through stubbles, plant debris and paddy soil	USA, Japan	Oster, 1992; Lanoiselet et al., 2002; Kimiharu et al., 2004; Hu et al., 2008
*Rhizoctonia oryzae, Rhizoctonia oryzae-sativae*	Basidiomycota, Corticiales	*Waitea circinata, Ceratobasidium oryzae-sativae*	Overwintering through stubbles, plant debris and paddy soil	Brazil, Japan	Prabhu et al., 2002; Kimiharu et al., 2004; Lanoiselet et al., 2007; Chaijuckam and Davis, 2010
**Bacteria**					
*Pseudomonas fuscovaginae*	Gamma proteobacteria	–	Highlands	31 countries	Miyajima et al., 1983; Zeigler and Alvarez, 1987; Flamand et al., 1996; Batoko et al., 1997
*Pseudomonas syringae*	Gamma proteobacteria	–	Ubiquitous epiphytic plant pathogen originally linked to aquatic systems	Hungary, Australia	Zeigler and Alvarez, 1990; Morris et al., 2013
*Pseudomonas palleroniana*	Gamma proteobacteria	–	–	La Réunion (France), Cameroon and Madagascar	Gardan et al., 2002
*Pseudomonas* sp.	Gamma proteobacteria	–	Ubiquitous	Cambodia, Philippines	Cottyn et al., 1996a,b; Cother et al., 2010; Patel et al., 2014
*Pantoea ananatis*	Gamma proteobacteria	–	Facultative pathogen	Australia, the Philippines, South Korea	Cottyn et al., 2001; Cother et al., 2004; Sinn et al., 2011; Choi et al., 2012; Cray et al., 2013
*Burkholderia glumae*	Beta proteobacteria	–	Adaptability to various habitats	USA	Sayler et al., 2006; Nandakumar et al., 2009; Ham et al., 2011; Paganin et al., 2011; Kim et al., 2014
*Burkholderia gladioli*	Beta proteobacteria	-	Adaptability to various habitats	USA	Nandakumar et al., 2009; Paganin et al., 2011
*Acidovorax oryzae*	Beta proteobacteria	*Pseudomonas avenae, Acidovorax avenae* subsp. *avenae*	Transmission by rain, wind and seeds	Philippines	Cottyn et al., 1996b; Schaad et al., 2008, Liu et al., 2012

The context of an increasing world population with shrinking natural resources imposes to adopt sustainable production methods, responding to the food demand but also using efficiently and sustainably key resources (Savary et al., 2000; Mew et al., 2004b). The development of sound control practices against rice sheath rot is hampered by the fact that this disease is poorly understood. This review would like to contribute in filling the rice sheath rot missing information gap. It explores the available information on the following aspects: causal agents and symptoms, host range, physiological and biochemical impact, virulence factors, synergism and interactions among causal factors, ecology of causal agents, epidemiology and impact, geographical distribution and relationships with farming systems and control methods. In this review, more emphasis will be put on rice sheath rot symptoms caused by *S. oryzae, Fusarium* sp., and *P. fuscovaginae*, since they are considered to be the most important rice sheath rot pathogens (**Table 2**).

**Table 2:** Main characteristics of the major rice sheath rot pathogens.

Pathogen	Survival	Host range	Most susceptible plant stage	Dissemination	Reproduction	Relevant metabolites	Reference
*Sarocladium oryzae*	Seeds, plant residues, soil, water	Weeds, bamboo, sedge	After booting stage	Wind, rain, insects, mites	Aseptate conidia	Helvolic acid, cerulenin	Pearce et al., 2001; Ghosh et al., 2002; Ayyadurai et al., 2005
*Fusarium fujikuroi*	Seeds, plant residues, soil		All stages	Wind, rain	Macro- and microconidia, no chlamydospores	Fumonisins (low levels in some strains), gibberellins, moniliformin	Abbas et al., 1999; Wiemann et al., 2013
*Fusarium proliferatum*	Seeds, plant residues, soil	Wide host range	All stages	Wind, rain	Macro- and microconidia, no chlamydospores	Fumonisins (high levels), moniliformin	Abbas et al., 1999
*Fusarium verticillioides*	Seeds, plant residues, soil	Wide host range	All stages	Wind, rain	Macro- and microconidia, no chlamydospores	Fumonisins (high levels)	Wulff et al., 2010; Wiemann et al., 2013
*Pseudomonas fuscovaginae*	Seeds, epiphytically and endophytically on rice	Wild and cultivated Gramineae	Seedling and booting stages	Wind, rain	Bacterial cells	Fuscopeptin, syringotoxin	Ballio et al., 1996; Flamand et al., 1996; Batoko et al., 1998

## *Sarocladium Oryzae:* The Major Fungal Rice Sheath Rot Pathogen

### Pathogen Description and Symptoms

*Sarocladium oryzae* was originally described as *Acrocylindrium oryzae*, the first organism to be associated with rice sheath rot symptoms isolated in Taiwan in 1922 (Mew and Gonzales, 2002). The genus *Sarocladium* was established in 1975 (Gams and Hawksworth, 1975) and currently encompasses 16 species including plant pathogens, saprobes, mycoparasites, endophytes, and potential human pathogens (Giraldo et al., 2015). The genus belongs to the order of the Hypocreales in the Phylum *Ascomycota*. *S. attenuatum* was originally described as a distinct species causing rice sheath rot, but is nowadays considered as a synonym of *S. oryzae* (Bridge et al., 1989). Bills et al. (2004) showed that also the cerulenin producing fungus *Cephalosporium caerulans* is conspecific with *S. oryzae*.

**FIGURE 1:**
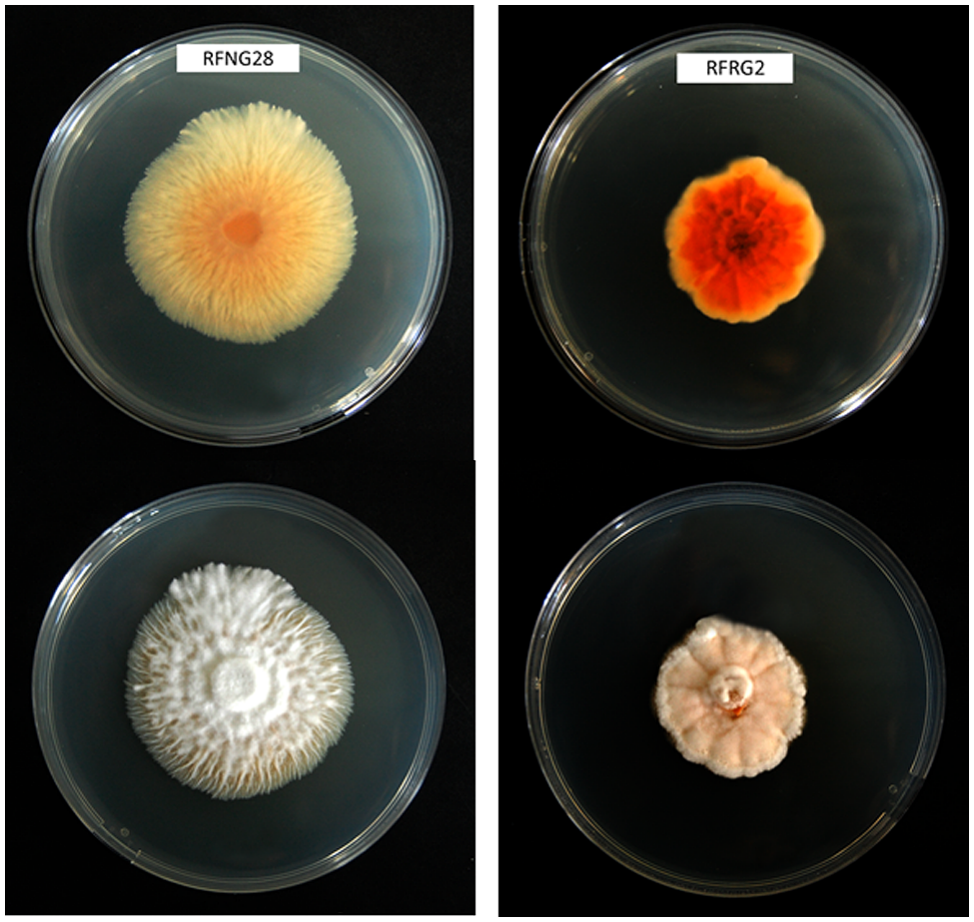
**Morphology of two different *Sarocladium oryzae* isolates from Rwanda on PDA medium after 14 days of growth at 28°C.** Top is reverse view, bottom is front view.

**FIGURE 2:**
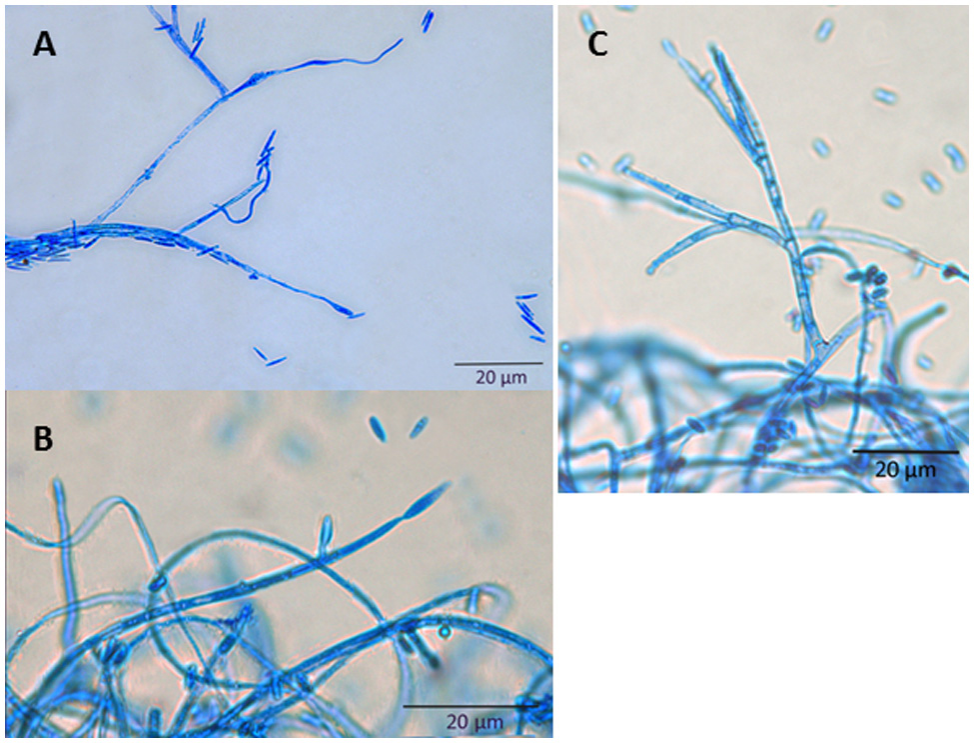
**Microscopy of *S. oryzae* grown on PDA medium.** All structures were stained with lactophenol blue. **(A)** Conidia; **(B)** Conidiogenous cell; **(C)** Aerial conidiophores.

*Sarocladium oryzae* grows slowly (about 2.5 mm/day on potato dextrose agar at 28°C) and produces a sparsely branched white mycelium. The colony reverse of isolates obtained from rice is generally orange (see **Figure 1**). Conidiophores can be simple or branched. Conidia are cylindrical, aseptate, and hyaline, 4–7 × 1–2 μm in size, and arranged in slimy heads (**Figure 2**).

**FIGURE 3:**
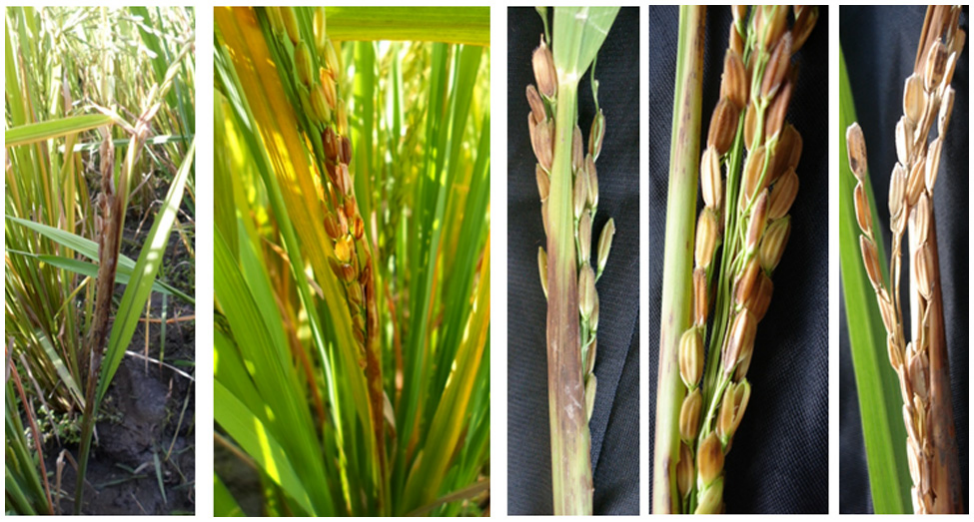
**Rice sheath rot symptoms caused by *S. oryzae* (photos M. Höfte)**.

The major symptoms describing rice sheath rot caused by *S. oryzae* are the following, according to Ou (1985): the rot occurs on the uppermost leaf sheaths enclosing the young panicles; the lesions start as oblong or somewhat irregular spots, 0.5–1.5 cm long, with brown margins and gray centers, or they may be grayish brown throughout; they enlarge and often coalesce and may cover most of the leaf sheath; the young panicles remain within the sheath or only partially emerge; an abundant whitish powdery growth may be found inside affected sheaths and young panicles are rotted. *S. oryzae* infection results in chaffy, discolored grains, and affects the viability and nutritional value of seeds (Sakthivel, 2001; Gopalakrishnan et al., 2010). The major symptoms of rice sheath rot incited by *S. oryzae* are presented in **Figure 3**.

### Epidemiology

In general, *S. oryzae* is present in all rice-growing countries worldwide, being very common in rainy seasons (Mew and Gonzales, 2002). It has so far been reported in the following countries (CABI, 2007): Bangladesh, Brunei Darussalam, China, India, Indonesia, Japan, Malaysia, Nepal, Pakistan, Philippines, Saudi Arabia, Sri Lanka, Tajikistan, Thailand, Uzbekistan, Vietnam, Burundi, Cameroon, Côte d’Ivoire, Gambia, Kenya, Madagascar, Niger, Nigeria, Senegal, Tanzania, Mexico, USA, Argentina, Brazil, Venezuela, and Australia. *S. oryzae* is mostly found in lowland environments (Pearce et al., 2001), and hot and humid weather favors the disease (Sakthivel, 2001). Sharma et al. (1997) stated that *S. oryzae* infections in Nepal were found below 1250 m. Temperatures of 20–30°C and relative humidity in the range of 65–85% favor sheath rot development (Sakthivel, 2001).

**FIGURE 4:**
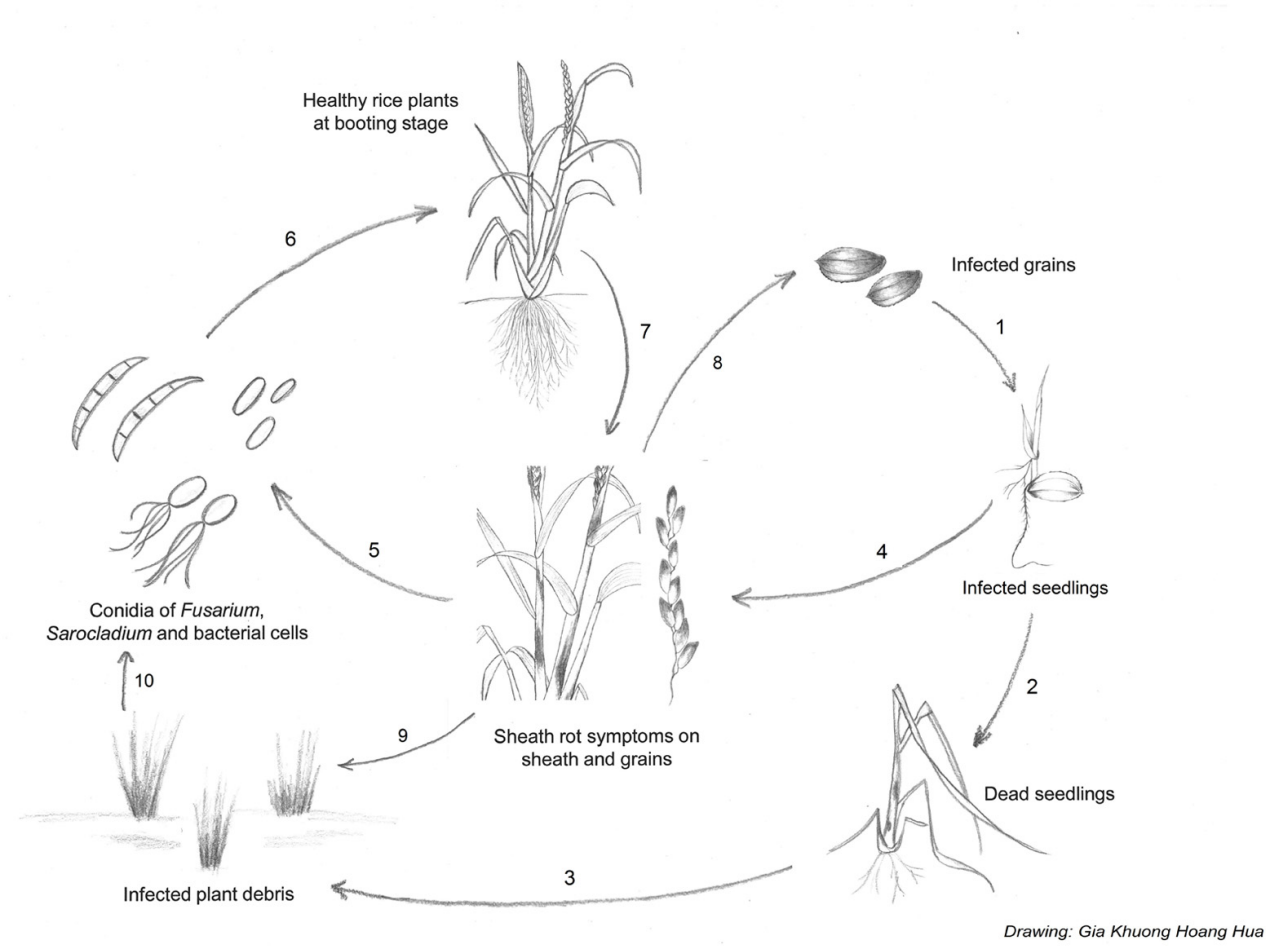
**Disease cycle of sheath rot caused by *S. oryzae, Fusarium* sp. or *Pseudomonas fuscovaginae*.** Sheath rot pathogens are seed-transmitted, resulting in infected seedlings (1). Infected seedlings can die (2) resulting in infected plant debris (3) or survive. *P fuscovaginae* can colonize the whole plant as an endophyte or survive epiphytically and infect the inflorescences at booting stage. The seedling transmission of the fungal pathogens is less well understood (4). Secondary infections result from conidia or bacterial cells released from infected plants (5). Conidia or bacterial cells are spread by wind or rain to healthy plants. Plants at booting stage are especially susceptible to infection. In the case of *S*. *oryzae*, insects and mites can also spread conidia and facilitate infection by creating wounds (6). Rot occurs on the sheath enclosing the young panicles; grains on affected tillers become chaffy and discolored. Grains infected with *Fusarium* sp. can become contaminated with mycotoxins (7). Pathogens can spread to new field via contaminated grains (8). After harvest, infected plant debris will remain in the field (9) serving as inoculum for the next growth cycle (10).

The pathogen survives in infected seeds, plant residues (straw, stubble), but also in soil, water or weeds when environmental conditions are favorable. Plants at various growth stages can be affected; the fungus enters through stomata or wounds, and is most destructive after booting stage but also infects other growth stages (Pearce et al., 2001). The entry of *S. oryzae* in the plant is facilitated mostly by insect and mite damage or the weakening of the plant by other pathogens (Pearce et al., 2001). Secondary infections may be wind-borne through injured tissues. Less is known about the seed-borne disease transmission. Caused yield losses are variable from 20 to 85%, depending on the pathosystem conditions (Sakthivel, 2001), (**Figure 4**).

The main host of *S. oryzae* is rice but the pathogen has also been reported as the causal agent of bamboo blight in Bangladesh and India. Bamboo isolates, however, show less infra-population variation than rice isolates (Pearce et al., 2001). *S. oryzae* has also been isolated from grasses and sedges growing in association with rice.

### Pathogenicity Determinants

**FIGURE 5:**
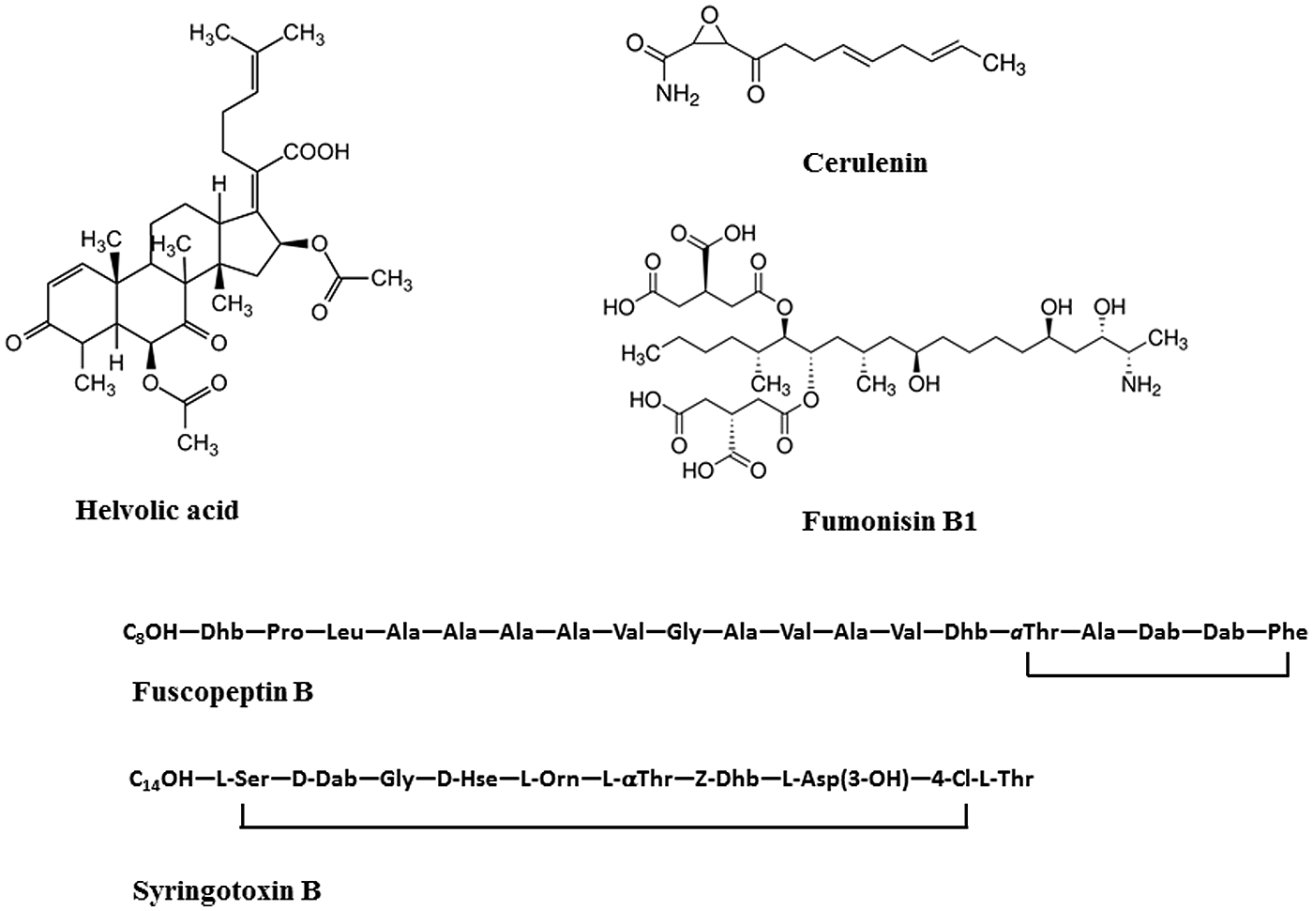
**Structures of toxins produced by rice sheath rot causing agents.** Helvolic acid and cerulenin are produced by *S. oryzae;* Fumonisin B1 is produced by *Fusarium* sp., Fuscopeptin B and syringotoxin B are produced by *P. fuscovaginae.*

Helvolic acid and cerulenin are described as the major secondary metabolites of *S. oryzae* (Ghosh et al., 2002; Ayyadurai et al., 2005), (**Table 3**, **Figure 5**). Artificial inoculation of those metabolites to host plants reproduced the sheath rot symptoms. Infiltration of rice tissues with cerulenin and helvolic acid leads to electrolyte leakage proportional to the susceptibility to rice sheath rot (Sakthivel et al., 2002). Tschen et al. (1997) reproduced *S. oryzae* symptoms on rice seeds, growth retardation and chlorosis, by dipping them in a solution of helvolic acid. Helvolic acid is a tetracyclic triterpenoid that interferes with chlorophyll biosynthesis (Ayyadurai et al., 2005). This compound is also produced by various other fungi including the opportunistic human pathogen *Aspergillus fumigatus*, the entomopathogenic fungus *Metarhizium anisopliae* and by endophytic fungi. Cerulenin is a hexaketide amide that inhibits polyketide synthesis by inhibiting the malonyl-ACP:acyl-ACP condensation step as well as fatty acid synthesis (Omura, 1976), (**Table 3**).

**Table 3:** Main toxins involved in rice sheath rot disease.

Microbial toxin	Producing sheath rot pathogen	Other producing organisms	Class	Mode of action	Symptom on plants	Other activities
Helvolic acid	*Sarocladium oryzae*	*Metarhizium anisopliae, Aspergillus* sp., *Pichia guilliermondii, Alternaria* sp.	Steroid	Interference with chlorophyll biosynthesis	Chlorosis	Antibacterial activity
Cerulenin	*Sarocladium oryzae*	Not known	Hexaketide amide	Inhibitor of fatty acid synthetases, interference with flavonoid biosynthesis	Necrosis, growth inhibition	Antibacterial and antifungal activity
Fumonisin B	*Fusarium proliferatum, F. verticillioides, F. fujikuroi*	Other *Fusarium* sp., *Aspergillus niger, Tolypocladium* sp., *Alternaria alternata*	Polyketide	Inhibitor of sphingolipid biosynthesis	Necrosis, growth inhibition	Human and animal toxin
Syringotoxin	*Pseudomonas fuscovaginae*	*Pseudomonas syringae* pv. *syringae*	Cyclic lipopeptide	Interference with ATPase pumps in plasma membrane	Necrosis	Antifungal activity
Fuscopeptins	*Pseudomonas fuscovaginae*	Not known	Cyclic lipopeptide	Form channels in plasma membranes	Necrosis	Antimicrobial activity

Though the most described virulence factors of *S. oryzae* are helvolic acid and cerulenin, the fungus also produces cellulolytic, proteolytic, pectinolytic, and oxidative enzymes that play a role in pathogenicity (Joe and Manibhushanrao, 1995; Pearce et al., 2001). Gopalakrishnan et al. (2010) observed a pronounced decrease in sugar, starch and protein and an increase in phenol content in rice seeds infected with *S. oryzae.* This probably explains why infected grains are chaffy and germinate poorly.

### Interactions with Other Diseases and Pests

Experimental tests have shown that *S. oryzae*, by the production of toxins, like cerulenin, limits the development of other fungi and emerges as the major pathogen (Gnanamanickam and Mew, 1991; Silva-Lobo et al., 2011). Gnanamanickam and Mew (1991) observed that the antibiotic properties of cerulenin extracted from *S. oryzae* block the development of many rice stem-attacking fungi, like *Sclerotium oryzae, Gaeumannomyces graminis* var. *graminis, Magnaporthe oryzae*, and *Rhizoctonia solani*. In this context it is interesting to notice that cerulenin has been reported to inhibit melanin biosynthesis in *Colletotrichum lagenarium* (Kubo et al., 1986). DHN (=1,8 dihydroxynapthalene)-melanin in fungi is synthesized by a polyketide pathway which starts from malonyl-CoA which is converted to the first detectable intermediate of the melanin pathway 1,3,6,8-tetrahydroxynapthalene via a polyketide synthase. DHN-melanin is an important virulence factor in several pathogenic fungi including *M. oryzae* and *G. graminis* var. *graminis* (Henson et al., 1999). In addition, helvolic acid has strong antibacterial activities mainly against Gram-positive bacteria (Tschen et al., 1997). This could explain why in many situations *S. oryzae* emerges as the major pathogen.

Initial work on sheath rot was done in India, and Amin et al. (1974) already realized the disease complexity, as the causal agent was already thought to be associated with stem borers. A study on four groups of insects: green leaf hopper, brown plant hopper, mealy bugs, and earhead bugs showed that brown plant hoppers and mealy bugs fed on rice infected with *S. oryzae* carry the fungus on their body and can transmit it to healthy plants (Gopalakrishnan et al., 2009). Some of the *S. oryzae* effects like sterility result from its synergism with a mite *Steneotarsonemus spinki* (Ou, 1985; Karmakar, 2008; Hummel et al., 2009). It was observed that wounding of plants facilitated their infection by *S. oryzae* and most of the infected plants proved also to be attacked by stem borers and from time to time by yellow dwarf virus (Ou, 1985). The fact that spraying a spore suspension of *S. oryzae* on earhead bug (*Leptocorisa acuta*)-infected rice tillers results in the development of rice sheath rot disease symptoms in 12 days (Lakshmanan et al., 1992) shows that the entry of *S. oryzae* in rice plants might be facilitated. Sakthivel (2001) realized that the infection occurs after the plant has been weakened by other diseases and stem borer infestation.

Bacterial sheath brown rot, caused by *P. fuscovaginae*, may occur together with sheath rot caused by *S. oryzae*. Other factors that have been associated with *S. oryzae* include rice tungro virus (Venkataraman et al., 1987) and *Fusarium* sp. (Sakthivel, 2001).

### Control Methods

*Sarocladium oryzae* is controlled by sanitary, chemical, and biological measures.

Sanitary control methods involve the following practices (Sakthivel, 2001): using healthy seeds since the disease is referred to as being seed-borne; limiting insect population in rice fields as they are involved in disease transmission; avoiding densely planting as this predisposes plants to fungal attacks; avoiding heavy doses of nitrogen fertilizers; increasing potassium content in the fertilizer formula for reducing the disease impact, as more potassium causes more phenol production; adopting different cultural practices for limiting the disease attack impact: field sanitation, crop residue management, control of weeds, etc.

Various fungicides have been used to control sheath rot but as they cannot kill the fungus inside the glumes, their efficacy is limited (Sakthivel, 2001). Other control tests combined fungicides with insecticides and gave better results (Lakshmanan, 1992). Foliar spray of micronutrients is also said to reduce disease incidence and increase grain yield (Sakthivel, 2001). Some plant extracts are reported to be effective against the disease: neem, pungam oil, and rubber cakes (Narasimhan et al., 1998; Sakthivel, 2001).

The use of biological control agents may have potential (Sakthivel and Gnanamanickam, 1987; Mew et al., 2004a). Many pseudomonads can act efficiently for controlling *S. oryzae*, by favoring antagonism, for example through the inhibition of fungal development as do some *P. fluorescens* strains, or by inducing systemic resistance (Saravanakumar et al., 2009).

Breeding for resistance to sheath rot seems the best option, but it is limited by its multiple causal agents. High-yielding nitrogen-responsive rice cultivars are highly susceptible to sheath rot. Resistance to *S. oryzae* has been identified in tall rice varieties (Amin, 1976). Hemalatha et al. (1999) developed a method of screening for resistance against *S. oryzae* based on a crude toxin preparation and Lakshmanan (1993b) went further by screening for resistance against *S. oryzae* and one of its vectors, the rice mealy bug. The screening of resistance against *S. oryzae* that was developed by Amin (1976) does not seem to have been continued. Ayyadurai et al. (2005) analyzed *S. oryzae* isolates from North East and South India and found a high variability in pathogenicity, phytotoxic metabolite production, and RAPD band patterns. This variability should be taken into account in breeding efforts.

## *Fusarium Fujikuroi:* A Species Complex Associated with Rice Sheath Rot

### Pathogen Description and Symptoms

Sheath rot in rice has also been associated with *Fusarium* sp. belonging to the *F. fujikuroi* complex. The *F. fujikuroi* complex largely corresponds to the Section *Liseola*, established by Wollenweber and Reinking (1935), in which Nelson et al. (1983) recognized four species (including *F. moniliforme* and *F. proliferatum)*, but also accommodates certain species originally classified in other *Fusarium* sections. Progress in molecular taxonomy has shown that there are around 50 species in the *F. fujikuroi* complex and the number keeps increasing (reviewed in Kvas et al., 2009). The complex is currently divided in three large clades, the African clade, the Asian clade and the American clade. The main organisms associated with rice are *F. verticillioides* from the African clade and the closely related species *F. proliferatum* and *F. fujikuroi* from the Asian clade.

Abbas et al. (1998) described rice sheath rot symptoms caused by *F. proliferatum* as follows: blanked or partially blanked panicle with reddish-brown to off-white florets or kernels are often covered with a white to pinkish white powder consisting of microconidia and conidiophores of *F. proliferatum*; the flag leaf sheath develops a rapidly enlarging lesion, first dull to dark brown and later off-white to tan with a reddish brown border, that eventually encompasses the entire sheath and may result in the death of the leaf blade; lower leaf sheaths may eventually develop lesions as well, but rarely more than two leaf sheaths show symptoms; and a dense white to pinkish white powder consisting of microconidia and conidiophores of *F. proliferatum* covers the sheath lesions, especially evident during humid periods.

### Epidemiology

Rice-pathogenic *Fusarium* species, because of their high diversity, are ubiquitous in nature (Park et al., 2005). Symptoms of rice sheath rot caused by any of the members of the *F. fujikuroi* species complex are widespread due to their large variability and at least one of their members is found in any part of the rice-growing world. The different species of *Fusarium* forming the *F. fujikuroi* complex (mainly *F. fujikuroi, F. verticillioides*, and *F. proliferatum*) cause various symptoms on different plant parts and are responsible of yield losses of 40% in Nepal (Desjardins et al., 2000) and even up to 60% in Korea (Park et al., 2005).

*Fusarium proliferatum*, which is pathogenic to rice, also attacks some other plants of the *Poaceae* family. *F. proliferatum* is widespread and its hosts vary from maize to mango (Leslie et al., 2007), including chestnut (Kushiro et al., 2012), and banana (Li et al., 2012). As the organisms causing rice sheath rot have many hosts, they can easily find alternate hosts in the environment, especially weeds.

*Fusarium* sp. are seed-transmitted and at maturity, infected grains contain mycotoxins (Wulff et al., 2010) (**Figure 4**). *F. fujikuroi* was one of a number of microbes isolated from the surface of rice seeds; highest levels of microbes were recorded at harvesting. *F. fujikuroi* survived for up to 26 months in infected grains and 28 months in dried stubble of certain rice cultivars. The fungus was detected for up to 10 and 13 months, respectively, in unsterilized and sterilized soils that were infected with fungal propagules (Sunder and Satyavir, 1998). *F. proliferatum* can survive in infected grains even when they are preserved in stressing conditions. In fact, Kushiro et al. (2012) could recover *F. proliferatum* in grains preserved at 4–5°C for 6 months. In normal conditions, the survival is longer.

### Pathogenicity Determinants

Two categories of metabolites are involved in pathogenicity and interaction with plants, gibberellins and mycotoxins. According to Wulff et al. (2010), only strains of *F. fujikuroi* were able to produce gibberellin A and these strains cause abnormal elongation of rice plants, the so-called bakanae disease. Main species producing mycotoxins, like fumonisin B (**Table 3**, **Figure 5**), have been reported to cause rice sheath rot (Wulff et al., 2010). Fumonisins are linear, polyketide-derived molecules that are also known as major mycotoxins that pose health risks to humans and animals. *F. proliferatum* is among the largest producers of fumonisins and is often associated with rice sheath rot (Abbas et al., 1999; Kushiro et al., 2012; Quazi et al., 2013). In addition, *F. verticillioides* strains are notorious fumonisin producers (Wulff et al., 2010). Isolates belonging to various other related *Fusarium* species have been shown to produce fumonisins (**Table 3**). Fumonisin biosynthetic genes have also been found in more distantly related fungi such as *Aspergillus niger* and *Tolypocladium* sp. The evolution of the fumonisin gene cluster in *Fusarium* is complex and not adequately represented by the species phylogeny. It is hypothesized that a combination of multiple horizontal gene transfer, cluster duplication and loss has shaped the current distribution of the fumonisin gene cluster (Proctor et al., 2013). The role of fumonisins in the ecology and pathology of *Fusarium* is poorly understood. Abbas et al. (1998) observed that the concentration of fumonisins coincides with the intensity of sheath and panicle symptoms in rice plants showing sheath rot under *Fusarium* attacks. Toxins are apparently concentrated in the external grain part since their concentration in the grain reduced 75–80% after hulling. One of the major fumonisins, FB1, is conceived as a virulence factor in *Fusarium*-induced diseases in plants (Glenn et al., 2008). FB1 inhibits ceramide synthase (Williams et al., 2007), an enzyme involved in sphingolipid biosynthesis in both animals and plants. This has numerous consequences on the cell physiology and results in increased cell death and inhibition of plasma membrane ATPases (Gutiérrez-Nájera et al., 2005). Members of the *F. fujikuroi* complex also produce a variety of other mycotoxins, including moniliformin. It has been shown that *F. proliferatum* isolates from field samples of rice with Fusarium sheath rot disease are capable of producing both fumonisins and moniliformin in culture. Both mycotoxins were also detected in naturally contaminated rice samples (Abbas et al., 1999). The phytotoxicity of moniliformin is well documented (Abbas et al., 1995). Moniliformin was shown to arrest mitosis of maize root meristematic cells at the metaphase stage (Styer and Cutler, 1984). The factors triggering the infection of *F. proliferatum* to rice plants still need to be further investigated (Kushiro et al., 2012). Genome sequencing revealed the presence of a wide variety of secondary metabolite gene clusters in *F. fujikuroi* and *F. verticillioides*, including clusters for bikaverin, fusarubins, fusarins, fumonisins, and fusaric acid. Beauvericin and gibberellin gene clusters, however, were only present in *F. fujikuroi* (Wiemann et al., 2013).

### Interactions with Other Diseases and Pests

There are reports of association of *Fusarium* sp. with *S. oryzae* in the rice sheath rot disease (Sakthivel, 2001). Islam et al. (2000) realized that in many seeds, numerous organisms are detected at the same time as *Fusarium*, including *Alternaria padwickii, Curvularia* sp., *S. oryzae, Magnaporthe oryzae, Bipolaris oryzae*, and *Microdochium oryzae*.

### Control Methods

Cultural and sanitary methods to control of rice sheath rot caused by *Fusarium* sp. include the use of clean seeds and water to separate light weight seeds (IRRI, 2015b). In chemical control, some fungicides are very effective against the fungus: thiophanate-methyl, benomyl, difenoconazole, penconazole (Ilyas and Iftikhar, 1997), and seed treatment is also advised. Seed dressing with antagonistic yeasts in combination with thermotherapy appears to be a promising strategy to control *F. fujikuroi* on rice seeds (Matić et al., 2014). Soil inoculation with the fungus *Talaromyces* sp. isolate KNB422 controlled seed-borne diseases on rice seedlings including *F. fujikuroi* as effectively as chemical pesticides (Miyake et al., 2012).

## Other *Fusarium* Sp. Associated with Rice Sheath Rot

*Fusarium graminearum* is grouped in the *F. graminearum sambucinum* complex (Aoki et al., 2014) and is pathogenic to many plants, mainly causing wheat head blight (Goswami and Kistler, 2004; Leplat et al., 2012). It has also been reported to cause sheath rot on rice (Singh and Devi, 1990; Naeimi et al., 2003).

*Fusarium equiseti* belongs to the *Fusarium incarnatum-equiseti* species complex (Aoki et al., 2014) and has been mainly reported as a pathogen for barley (Marín et al., 2012) and wheat (Castellá and Cabañes, 2014). It was also isolated from rice stem tissues (Fisher and Petrini, 1992).

*Fusarium oxysporum* forms its own group according to the phylogenetic relationships of key *Fusarium* species (Aoki et al., 2014). Though most of the time it has been associated only to vascular diseases and not to *Poaceae* plants (Agrios, 2005), it has been isolated from rice plant tissues (Fisher and Petrini, 1992; Abbas et al., 1995; Desjardins et al., 2000) and is pathogenic on young rice plants (Prabhu and Bedendo, 1983; Fisher and Petrini, 1992). Some *F. oxysporum* isolates are known to produce fumonisins (Proctor et al., 2008), but whether isolates associated with rice sheath rot symptoms produce these mycotoxins has not been tested.

## Related Fungal Diseases

*Cochliobolus lunatus* causes black kernel disease on rice and has been identified as the causal agent of rice sheath rot in India and Bangladesh (Lakshmanan, 1992, 1993a; Shamsi et al., 2003). There are no extensive studies on its pathogenesis on rice, but its virulence is attributed to the methyl 5-(hydroxymethyl) furan-2-carboxylate (M5HF2C) toxin (Liu et al., 2009; Gao et al., 2015).

*Gaeumannomyces graminis* var. *graminis* (Syn.: *Ophiobolus oryzinus*) causes crown sheath rot or black sheath rot on rice (Walker, 1972; Frederick et al., 1999; Peixoto et al., 2013) and its virulence is linked to the production of DHN-melanin (Frederick et al., 1999).

*Sclerotium hydrophilum* was recognized as an agent of sheath leaf necrosis by Lanoiselet et al. (2002). The fungus was isolated from infected rice sheaths and was shown to cause rice leaf sheath disease. But *Sclerotium hydrophilum* is not the only sclerotial disease of rice. *Rhizoctonia fumigata, R. oryzae-sativae, R. oryzae*, and *R. solani* are reported to induce the same symptoms as *Sclerotium hydrophilum* leaf sheath disease (Kimiharu et al., 2004). The damage caused by all these diseases is high when they reach the top leaf sheath of the plant. The symptoms of all these diseases are pronounced at the heading stage and increase as the plant matures. Most of the time, the rice sclerotial diseases cause overlapping symptoms in places where sheath blight caused by *R. oryzae* frequently occurs, although their pathogenesis is different (Prabhu et al., 2002). These diseases have in common with *S. oryzae*, the most reported rice sheath rot pathogen, and other sheath rot agents that their symptoms are more pronounced in the reproductive stage and around physiological maturity (Oster, 1992). Also, in the description of the symptoms of *R. oryzae-sativae* (Syn: *Ceratobasidium oryzae-sativae*), Lanoiselet et al. (2007) mentioned classical sheath rot disease associated symptoms like the rotting of the culm and grain sterility.

The diseases caused by *Cochliobolus lunatus, Gaeumannomyces graminis, Sclerotium hydrophilum, R. fumigata, R. oryzae-sativae, R. oryzae, R. solani*, though they are closer to rice sheath rot agents in terms of symptomatology, will not be extensively covered in this review, considering that they have been primarily described based on plant parts different from the rice sheath.

## *Pseudomonas Fuscovaginae:* The Most Important Bacterial Pathogen Associated with Rice Sheath Rot

### Pathogen Description and Symptoms

**FIGURE 6:**
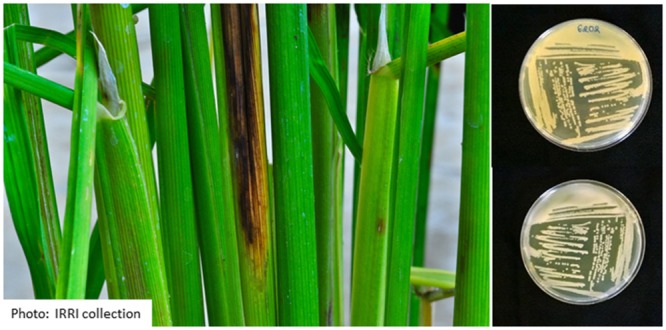
**Symptoms caused by *P. fuscovaginae* and morphology on King’s Medium B plates after 48 h of growth at 28°C (top is reverse side, bottom is front side)**.

Since its isolation in association with rice sheath rot in Japan (Tanii et al., 1976; Miyajima et al., 1983) and its identification as the causal agent of discoloration of rice sheaths, leaves and grains in Latin America (Zeigler and Alvarez, 1987), *P. fuscovaginae* is considered as the main bacterium causing rice sheath brown rot. It has been found on both the sheath and the glume (Cother et al., 2009). Zeigler and Alvarez (1987) stated that rice sheath brown rot, caused by *P. fuscovaginae* in Latin America, is characterized by the following features: longitudinal brown to reddish brown necrosis 2–5 mm wide extending the entire length of the leaf sheath and blade; affected sheaths enclosing the panicle may show extensive water-soaking and necrosis with poorly defined margins; glumes discolor before emerging from such panicles; grains on affected tillers may be completely discolored and sterile to nearly symptomless with only small brown spots. To these symptoms, the description by Cottyn et al. (1996a) adds the following features: a wide range of sheath and/or grain symptoms, varying from translucent to brown dots to brown blotches to brown streaks to a completely brown sheath, and/or clear to brown spots to brown blotches to completely dark discolored seeds. An illustration of bacteria-induced rice sheath rot is presented in **Figure 6**.

The genus *Pseudomonas* belongs to the subclass *Gammaproteobacteria* of the Gram-negative bacteria and currently comprises 144 species. Based on multilocus sequence analysis, *P. fuscovaginae* belongs together with *P. asplenii* to the *P. asplenii* subgroup as defined by Gomila et al. (2015). These two species are closely related and some authors consider them to be synonymous (Vancanneyt et al., 1996). The original description of *P. fuscovaginae* in Miyajima et al. (1983) is the following: the cells are aerobic, gram negative, non-spore-forming, rod-shaped with round ends, 0.5–0.8 × 2.0–3.5 μm. Cells occur singly or in pairs and are motile by means of one to four polar flagella. They oxidize glucose in oxidation–fermentation medium, and they produce a green fluorescent pigment, oxidase and arginine dihydrolase. Denitrification, β-glucosidase, pit formation on polypectate gel and growth at 37°C are negative. Characteristics that distinguish this species from other fluorescent pseudomonads which are positive for arginine dihydrolase and oxidase are its inability to utilize 2-ketogluconate or inositol.

Whole genome sequence analysis of various *P. fuscovaginae* strains has revealed that these pathogens do not form a single monophyletic group. At least two subgroups have been identified and strains from Madagascar, Japan, China, and Australia clustered separately from *P. fuscovaginae*-like strains from the Philippines (Quibod et al., 2015).

### Epidemiology

*Pseudomonas fuscovaginae* was first reported in literature as the pathogen responsible for rice sheath rot disease in cold and humid tropical highlands in Japan (Miyajima et al., 1983), Burundi (Duveiller et al., 1988), Madagascar (Rott et al., 1989), and Nepal (Sharma et al., 1997), but it was later found even in lowlands (Cottyn et al., 1996a). *P. fuscovaginae* is also associated with high rainfall (Sharma et al., 1997). The bacterium causes quantitative and qualitative losses (Zeigler and Alvarez, 1987). For losses in quality, symptomatic grains cannot be accepted in seed certification chains, mills accept them with a discount and they have a poor marketing value.

CABI (2007) reports the presence of *P. fuscovaginae* in 31 countries: Former Yugoslavia, Russian Federation, China, Indonesia, Japan, Nepal, Philippines, Burundi, Democratic Republic of Congo, Madagascar, Rwanda, Tanzania, Costa Rica, Cuba, Dominican Republic, El Salvador, Guatemala, Jamaica, Nicaragua, Panama, Trinidad and Tobago, Mexico, Argentina, Bolivia, Brazil, Chile, Colombia, Ecuador, Peru, Suriname, and Uruguay. Recently, the disease has been diagnosed in Australia (Cother et al., 2009).

The host range of *P. fuscovaginae* seems to be restricted to wild and cultivated *Gramineae* (Tanii et al., 1976; Miyajima et al., 1983).

*Pseudomonas fuscovaginae* is seed-transmitted and infected seedlings often die. When infection occurs at a later stage, the lower part of the sheath becomes brown and later on, the whole sheath becomes necrotic. The pathogenicity of *P. fuscovaginae* is expressed at grain, seedling and booting stage levels. *P. fuscovaginae* is able to colonize the whole plant as an endophyte (Adorada et al., 2015). If the seedling survives, *P. fuscovaginae* has an epiphytic life until the booting stage when it infects inflorescences, resulting in the formation of infected grains or the panicle abortion. The population of the bacterium is maintained at a low level from early growth stages up to the booting stage. The bacterium can survive epiphytically on the host plant with low pathogen population in the tissue and this explains how the disease can be seed-borne, but only express symptoms at the booting stage (Batoko et al., 1997) (**Figure 4**). This can also be linked to the fact that the booting stage and the reproductive phase in general, is the most stress-sensitive stage in the rice plant development (Fageria, 2007).

### Pathogenicity Determinants

Different phytotoxins are involved in the disease development. Batoko et al. (1997) could reproduce sheath brown rot symptoms by inoculating seedlings with toxins from bacteria. They concluded that toxins are an integral part of the plant-pathogen interactions in rice bacterial sheath rot. Flamand et al. (1996) found that a cell-free extract from *P. fuscovaginae* that could induce the same symptoms as *P. fuscovaginae* contained five peptidic compounds (A, B, C, D, and E) and two others (fuscopeptins A and B). Peptidic compound D is identical to syringotoxin, a lipodepsinonapeptide containing nine amino acids acylated by 3-hydroxytetradecanoic acid (**Table 3**, **Figure 5**) that is also produced by *P. syringae* pv. *syringae* pathogenic on citrus (Ballio et al., 1990). The structure of fuscopeptins was elucidated by Ballio et al. (1996). Fuscopeptins are lipodepsipeptides containing 19 amino acids. Fuscopeptin A is acylated by 3-hydroxyoctanoate while fuscopeptin B is acylated by 3-hydroxydecanoate (**Table 3**, **Figure 5**). Both compounds target the plasma membrane and inhibit H^+^-ATPase and act synergistically with syringotoxin (Batoko et al., 1998).

Toxins from *P. fuscovaginae* are non-host specific, the pathogen inducing disease symptoms on many plants of the *Poaceae* family in addition to rice (Miyajima et al., 1983), and have more detrimental effect on culm elongation (Batoko et al., 1997). The non-host specificity may also be justified by the symptoms induction by *P. fuscovaginae* on *Chenopodium quinoa* (Mattiuzzo et al., 2011), a plant belonging to the *Amaranthaceae* family. Toxins are immediately dissolved in the plant thus become difficult to recover (Batoko et al., 1997). Phytotoxin concentration increases at the booting stage of rice, which stimulate their large production by the bacterium. The capacity of the plant to detoxify the toxins plays a pivotal role and could constitute a starting point in breeding for resistance against *P. fuscovaginae.*

Patel et al. (2014) were able to isolate nine mutants of *P. fuscovaginae* via random Tn5 mutagenesis which showed altered virulence on rice. Besides mutants affected in phytotoxin production, also mutants in type IV pili biosynthesis, type VI secretion, arginine biosynthesis and sulfur metabolism were obtained indicating that these processes are also involved in pathogenicity on rice.

### Interactions with Other Diseases and Pests

Most of the time *P. fuscovaginae* was found together with *S. oryzae* in sheath rot diseased plants (Zeigler and Alvarez, 1987; Cottyn et al., 1996a).

### Control Methods

Some cultural and sanitation practices against *P. fuscovaginae* are indicated like burning farm remains: stubbles, ratoons; treatment of seeds by dipping them in water at 65°C before sowing (Zeigler and Alvarez, 1987); introducing rotation; checking the quality of seeds and as it is a seed-borne disease, using healthy seeds. Host plant resistance is also considered as an option. There are limited sources of resistance to rice sheath rot (Adorada et al., 2013), while this is a must in developing a control strategy against the disease. There are various methods that can be used for screening resistance and Adorada et al. (2013) suggested using the pin-prick method. About the chemical control, streptomycin, alone or in combination with oxytetracycline, can effectively control rice sheath rot (CABI, 2007).

## Other *Pseudomonas* Sp. Associated with Rice Sheath Rot

Besides *P. fuscovaginae*, a variety of other poorly characterized fluorescent pseudomonads have been associated with rice sheath rot since the 1950s. The first characterized sheath rot associated *Pseudomonas* was *P. oryzicola* (Klement, 1955). Later on it was decided that this pathogen is equivalent to *P. syringae* pv. *syringae* (Young et al., 1978). Besides *P. syringae* and *P. fuscovaginae*, various other pseudomonads have been consistently found in rice sheath rot related studies (Zeigler and Alvarez, 1987; Cottyn et al., 1996a,b; Cother et al., 2010; Saberi et al., 2013). Only a few of those other pseudomonads have been fully identified except by biochemical tests.

Zeigler and Alvarez (1987) attempted to put rice sheath rot-associated pseudomonads into groups, which were continued and named, based on BIOLOG features, by Cottyn et al. (1996a). In their work, they defined, based on the guidelines for the taxonomy of Proteobacteria, originally called purple bacteria (Woese, 1987), four main groups of *Gammaproteobacteria* associated with rice sheath rot and grain discoloration named after the representative species: *P. putida, P. aeruginosa, P. fuscovaginae*, and a composite group related to *P. marginalis, P. corrugata, P. fluorescens, P. aureofaciens*, and *P. syringae*. Also Saberi et al. (2013) concluded, based on biochemical tests, that sheath rot and grain discoloration caused by *Pseudomonas* sp. in Iran are related to *P. marginalis, P. putida*, and *P. syringae*.

The question whether these associated *Pseudomonas* sp. are really pathogenic on rice remains posed for many years. From the start, few species emerged as the most pathogenic compared to others which were causing some low levels of the disease. Zeigler and Alvarez (1987) already mentioning minor sheath and grain disorders caused by fluorescent pseudomonads, *P. fuscovaginae* being the principal causal agent. Gardan et al. (2002) isolated *P. palleroniana* from La Réunion (France), Cameroon, and Madagascar from healthy or necrotic rice seeds and from diseased tissue of leaf sheaths. The *P. palleroniana* isolates inoculated to rice seedlings were either non-pathogenic or weakly pathogenic. On the contrary, typical symptoms of bacterial sheath brown rot were induced by *P. fuscovaginae* strain CFBP3078, introduced in the experiment for comparison. This shows that among the pseudomonads found with rice sheath rot, there are differences in virulence and *P. palleroniana* is among the weakly pathogenic organisms.

However, caution is needed in the interpretation of the pathogenicity level for the different species of the pseudomonads associated with rice sheath rot. Cother et al. (2010) isolated a pseudomonad causing a widespread disease similar to sheath brown rot in Cambodia. This bacterium was related to *P. parafulva* and *P. fulva*, which belong to the *P. putida* group as defined by Gomila et al. (2015), and was clearly pathogenic on rice.

In the meantime, the taxonomy of pseudomonads has made important progress especially thanks to molecular identification method development. In a recently published classification of *Pseudomonas* genus, based on the Multilocus Sequence Analysis technique (MLSA), Gomila et al. (2015) defined 19 groups and subgroups. Most of the sheath rot associated pseudomonads probably belong to the *P. chlororaphis, P. fluorescens, P. asplenii* (=*P. fusovaginae*) subgroup or the *P. putida* group, though the groupings are difficult to define currently as many isolates have not yet been fully analyzed.

## Related Bacterial Diseases

*Pantoea ananatis*, considered globally as a facultative pathogen (Cray et al., 2013), was demonstrated as a sheath rot pathogen with typical symptoms of necrotic spots and discoloration on glumes and stems, indistinct chlorosis but with no water-soaking and its pathogenicity testing satisfied Koch’s postulates (Choi et al., 2012). The disease had previously been reported in the Philippines (Cottyn et al., 2001) and in Australia (Cother et al., 2004), but its importance, though it is reported to reduce the grain quality when it infects the glumes, was never assessed. It was only presumed to be low. Furthermore, in pathogenicity tests, Cother et al. (2004) recovered the pathogen from the plants that had not been inoculated, which prompted the hypothesis that the organism lives as an epiphyte and triggers disease symptoms when the plant is under physiological stress. Also Choi et al. (2012) linked the disease appearance to favorable environmental conditions.

*Burkholderia glumae* and *B. gladioli* are becoming important rice pathogens (Nandakumar et al., 2007). *B. glumae* (formerly *P. glumae*) was reported as the agent of rice grain discoloration in Latin America (Zeigler and Alvarez, 1989) after it had been reported as a grain rotter in Asia. It was later detected in North America, in association with *B. gladioli*, causing bacterial panicle blight (Nandakumar et al., 2009). The two pathogens, in addition to being seed-borne, can also be soil-borne (Nandakumar et al., 2008). Disease symptoms are observed at the sheath and grain levels. Though the disease is seed-borne, the presence of the bacteria in the sheath plays a capital role in the infection of the emerging panicle. Toxoflavin, a toxin produced by both species, is considered to be the main pathogenicity determining factor (Suzuki et al., 2004; Ura et al., 2006), while a lipase produced by *B. glumae* (Pauwels et al., 2012) and tropolone produced by *B. gladioli* (Wang et al., 2013) have also been implicated in pathogenicity.

*Acidovorax oryzae* (Schaad et al., 2008), formerly called *Pseudomonas avenae* and *Acidovorax avenae* subsp. *avenae* (Willems et al., 1992), causes bacterial brown stripe on rice (Shakya et al., 1985; Kadota et al., 1991; Song et al., 2004). Symptoms start as brown stripes at the bottom of the stems and frequently extend into the sheaths (Liu et al., 2012). This bacterium has consistently been detected in rice sheath rot related studies (Cottyn et al., 1996a,b; Cortesi et al., 2005; Cother et al., 2010). Recently the type IV pili assembly protein PilP has been implicated in the pathogenicity of *A. oryzae* on rice (Liu et al., 2012).

## Conclusion and Perspectives

Since rice sheath rot symptoms were first described in Taiwan in 1922 and attributed to *S. oryzae*, various reports of similar or related disease symptoms have been produced in different parts of the world. Rice sheath rot is now getting momentum as an emerging destructive rice disease but on which the scientific understanding is still limited.

There are three main species or complexes of organisms that can cause rice sheath rot: *S. oryzae*, the *F. fujikuroi* complex, and *P. fuscovaginae*, but there are many others that are reported to induce symptoms close to those of rice sheath rot. Interestingly, all three groups of major sheath rot causing pathogens produce phytotoxins that cause necrosis and can mimic the disease symptoms, which is probably the reason why they all cause similar looking disease symptoms. The principle that “everything is everywhere, but, the environment selects” (De Wit and Bouvier, 2006) applies to rice sheath rot; organisms that can potentially cause rice sheath rot are many and can be found everywhere nowadays, but the environment probably selects the ones that can adapt to the prevailing environmental conditions in a given area. This situation results in the overlapping of symptoms in the rice sheath rot disease complex (Johanson et al., 1998; Hu et al., 2008) especially at the rice reproductive stage, the most stress-sensitive phase in rice development (Fageria, 2007). There can be even synergism among the rice sheath rot-associated organisms or with arthropods or other groups of organisms. Due to changes in agriculture and in the society in general, like the developments in the farming systems and increased mobility in general, there are also changes in plant health problems, some diseases becoming more important than before, like rice sheath rot, which is now becoming a serious threat to rice production in many parts of the world.

It is proven that most sheath rot associated pathogens have an endophytic (latent) phase in their lifecycle, waiting for the plant to become stressed so that they can attack it (Fisher and Petrini, 1992). This phenomenon is not recent, it was observed since many years. Hsieh et al. (1977) attested the presence of *F. moniliforme* (now known as the *F. fujikuroi* complex) on plants without causing visible disease symptoms. New empirical data are needed about most of the organisms thought to be endophytic as some of them have pathogenic potential and are waiting for conducive conditions for attacking the plant. Factors governing the expression of the virulence are not yet clearly understood (Andrews and Harris, 2000). There is an urgent need of associating molecular, genetic and pathogenicity data for elucidating the role and interactions with endophytes given that at the plant level, the answer to pathogens and endophytes is the same (Andrews and Harris, 2000).

The large variability observed in rice sheath rot associated *Pseudomonas* and *Fusarium* genera is intriguing. It would be interesting to investigate whether the isolates in these two groups that can cause sheath rot have obtained phytotoxin-encoding gene clusters by horizontal gene transfer. At least in the case of fumonisins, it has been shown that the fumonisin gene cluster has spread among *Fusarium* sp. and related genera by a combination of horizontal gene transfer, cluster duplication and loss (Proctor et al., 2013). It should be tested whether the sheath rot causing *Fusarium* isolates all contain the fumonisin gene cluster or other phytotoxin encoding gene clusters. Horizontal gene transfer is also a widespread phenomenon in fluorescent pseudomonads (Silby et al., 2011) and it is known that many gene clusters for secondary metabolites, including cyclic lipopeptides, are located on genomic islands. Again, this could be systematically tested for *Pseudomonas* isolates associated with rice sheath rot.

Rice sheath rot has become a highly destructive rice disease with a high variability in yield loss levels varying from 20 to 85%. It is caused by many pathogenic agents varying depending on the area, grown varieties, prevailing environmental conditions, the farming system, other pests, etc. Not much progress has been achieved in the control of the disease, partly because the etiology of the disease is difficult to establish. For facing the disease, a better understanding about it is needed and this review is contributing in that aim. As rice sheath rot disease is complex by nature, its control strategy must be inspired by the Integrated Pest Management (IPM) approach. The solution remains site-specific. Limiting the number of potential pathogens harbored by the plant, making the plant environment less conducive to pathogen development, etc. should be the central elements in the control approach, which can be complemented by other methods, indicated according to the context. The IPM approach is particularly relevant now that there is a need for feeding and responding to the other needs of a constantly increasing population while the production must be conducted in a sustainable way, meaning that the overreliance on pesticide must leave the room to scientifically proven environmentally friendly crop protection practices.

## Conflict of Interest Statement

The authors declare that the research was conducted in the absence of any commercial or financial relationships that could be construed as a potential conflict of interest.
